# The application prospect of metagenomic next-generation sequencing technology in diagnosing suspected lower respiratory tract infections

**DOI:** 10.3389/fcimb.2025.1494638

**Published:** 2025-04-16

**Authors:** Wei Li, Mingming Zhao, Weiwei Wu, Gang Chen, Yanping Hang, Haixia Zheng, Zhenyun Gao, Jia Liu, Yuguo Zhao

**Affiliations:** 1 Department of Respiratory Medicine, Nanjing Lishui People's Hospital, Zhongda Hospital Lishui Branch, Southeast University, Nanjing, Jiangsu, China; 2 Department of Pulmonary and Critical Care Medicine, Nanjing Gaochun People’s Hospital, Nanjing, Jiangsu, China; 3 Dinfectome Inc., Nanjing, Jiangsu, China

**Keywords:** suspected lower respiratory tract infection, metagenomic next-generation sequencing, COPD, pathogen spectrum, resistance genes

## Abstract

**Objective:**

Lower respiratory tract infections present substantial diagnostic and therapeutic challenges, negatively impacting individual health. This study aims to utilize metagenomic next-generation sequencing (mNGS) technology to comprehensively explore the spectrum of pathogens, the detection of antibiotic resistance genes, and contributing factors associated with lung infections.

**Method:**

The mNGS data of 217 patients with suspected lung infections attending the Respiratory Department of Nanjing Lishui People’s Hospital and Gaochun People’s Hospital from September 2022 to September 2023 were retrospectively analyzed. The study assessed the pathogenic spectrum of lung infections and compared the performance of patients with mNGS results from conventional microbiological techniques (CMT).

**Results:**

The overall positivity rate of mNGS was 95.20%, demonstrating superior sensitivity (97.01% vs. 41.79%) and accuracy (75.56% vs. 56.67%) compared to CMT. Bacterial infections were the most prevalent, accounting for 60.76% of cases. And the most prevalent bacteria, fungus and virus were *Mycobacterium tuberculosis* (14.41%), *Candida albicans* (15.72%), and EB virus (14.85%), respectively. The primary resistance genes detected were tetM (17, 8.29%), mel (6, 2.93%), and PC1 beta-lactamase (blaZ) (3, 1.46%). Notably, TEM-183, PDC-5 and PDC-3 were exclusively detected in the Chronic Obstructive Pulmonary Disease (COPD) group. The multivariate binary logistic regression analysis revealed that there was no significant association between gender, presence of hypertension, or COPD with the type of infection in patients (*p*=0.679, *p*=0.229, *p*=0.345). However, the immune status was found to be statistically significant (*p*=0.009).

**Conclusion:**

With the guidance of mNGS, patients with suspected respiratory tract infections can rapidly and accurately establish a pathogenic basis for their conditions. mNGS effectively identify mixed infections, enrich the pathogen spectrum of lung infections, and provide a large and reliable information base for the clinical realization of targeted medication.

## Introduction

Respiratory infections remain a widespread concern worldwide due to their high morbidity, mortality and burden (Li and Nair, 2022; Bolek et al., 2023). Although there has been a general decline in age-standardized rates of morbidity, mortality, and disability-adjusted life years (DALY) in many regions (GBD 2019 LRI Collaborators, 2022), pneumonia continues to be the fourth most common cause of death (Cillóniz et al., 2020). Lower respiratory tract infection-related mortality has decreased dramatically as both the economy and medical circumstances have improved, with 370,000 fewer deaths occurred in 2021 compared to 2000, yet 2.5 million deaths remain (World Health Organization, 2020).

The main pathogenic microorganisms also differ between age groups and are frequently associated with fungal or viral infections (Liu et al., 2023), which in severe cases can lead to the development of sepsis (Rudd et al., 2020). Following the COVID-19 pandemic, there was a notable decrease in invasive respiratory infections caused by *Streptococcus pneumoniae, Haemophilus influenzae*, and *Neisseria meningitidis*, but this was consistent with the adoption of containment measures (Brueggemann et al., 2021). Subsequently, the incidence of influenza, respiratory syncytial virus (RSV), and other respiratory viruses increased (AlBahrani et al., 2024), accompanied by fluctuations in the monthly prevalence of pathogens (Nazareth et al., 2023).

In order to provide patients with more prompt and accurate treatment, early pathogen detection is crucial. Currently, culture is still considered the “gold standard” in clinical practice. Other conventional diagnostic techniques include smear microscopy, serology, and PCR (Walter and Wunderink, 2018), but these techniques frequently fall short of providing a prompt and thorough response, and the use of broad-spectrum antibiotics to stabilize a patient’s condition before the results of the infecting microorganisms are clear can negatively affect the patient’s subsequent diagnosis and treatment, particularly if the condition worsens and poses a threat to the patient’s health.

Metagenomic next-generation sequencing (mNGS) has emerged as a promising tool in the clinical management of infectious diseases. Its ability to provide objective extraction and detection of nucleic acids offers a significant advantage over traditional methods, such as PCR, by reducing specificity issues and enhancing sensitivity. This technology facilitates earlier and more accurate diagnosis, which can potentially improve patient outcomes and optimize the use of antimicrobial drugs for respiratory tract infections (Lv et al., 2024). As mNGS continues to evolve, its role in enhancing the management of respiratory infections is likely to expand, providing new opportunities for clinical practice.

## Materials and methods

### Patients and study design

**Figure 1:**
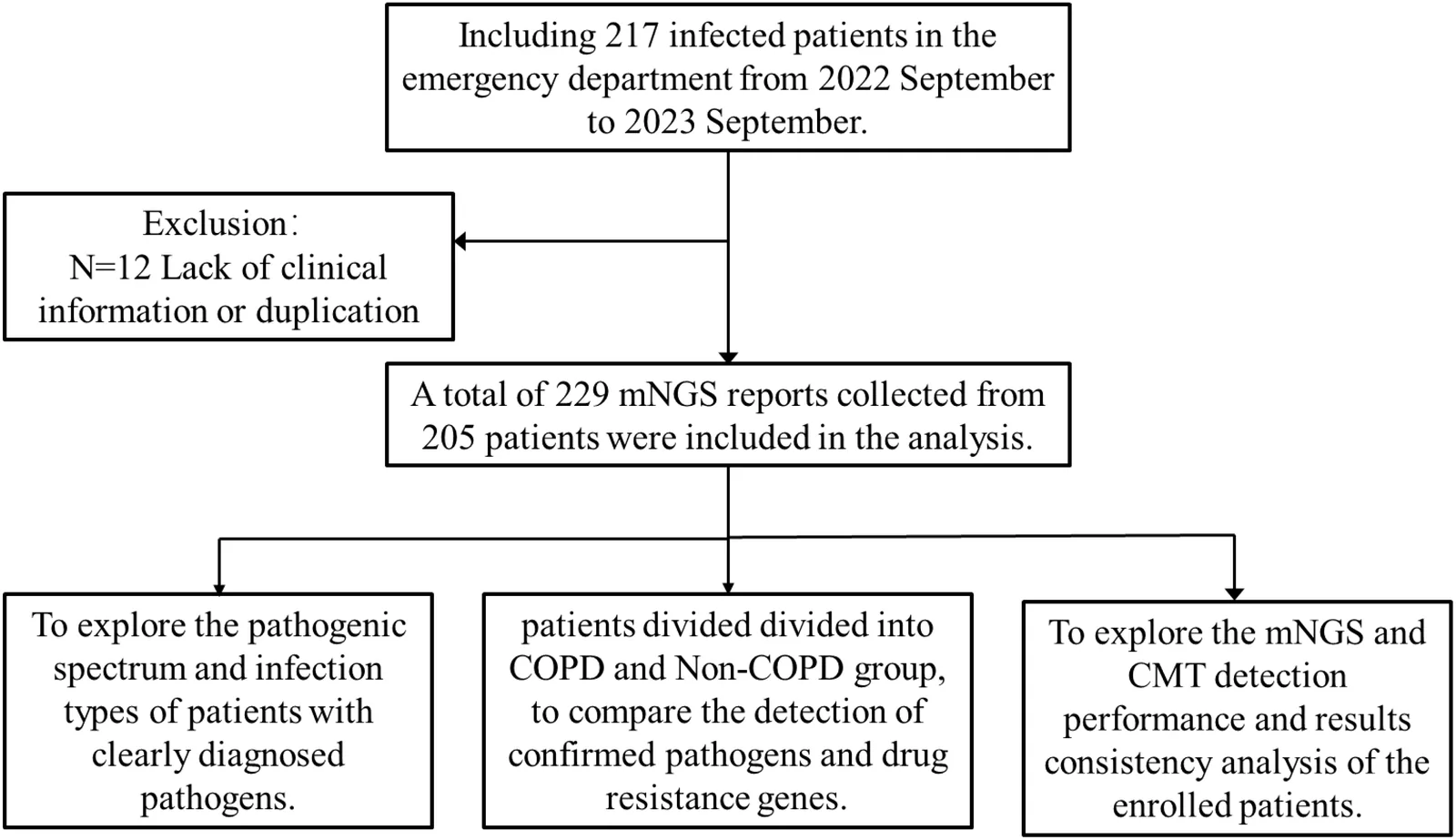
Patient enrollment work flow chart.

Retrospective inclusion was made for 217 patients with suspected lung infections who visited the respiratory departments of Nanjing Lishui People’s Hospital and Gaochun People’s Hospital between September 2022 and September 2023. Patients were not restricted by age or gender and agreed to undergo metagenomic next-generation sequencing for detection. 12 patients with missing clinical indicators or duplicated information were excluded, and finally, the clinical data of 205 patients, including information on age, gender, underlying diseases, laboratory results, and patient prognosis, were subjected to demographic and baseline characterization. And we analyzed the overall detection status of mNGS, compared the detection performance of 84 patients with traditional results (**Figure 1**).

### Sample collection

Depending on the patient, several sample types were obtained for this investigation, including pleural fluid, blood, and alveolar lavage fluid. Alveolar lavage fluid was collected from patients with suspected lung infections by an experienced clinician after obtaining informed consent for bronchoscopy. Patients were given intravenous and local anesthesia with 2% lidocaine. The most serious lesion was selected for lavage based on chest imaging, with the left lingual lobe or right middle lobe chosen in cases of diffuse lesions. Sterile normal saline was injected into the target bronchus in several equal amounts, and the fluid was then collected into a sterile test tube after the procedure was completed. Approximately 8-10 mL of blood sample was collected through a DNA negative pressure anticoagulation sampling tube. Blood sample was obtained using anticoagulation tubes filled under negative pressure conditions. Pleural fluid was obtained by a physician by percutaneous puncture or surgical means under sterile conditions. Samples were simultaneously analyzed by the Department of Pathology using conventional microbiological tests (CMTs) and sent to Dinfectome Inc (Nanjing, China) for pathogenic DNA testing.

### Conventional microbiological tests

The samples were subjected to a series of laboratory tests immediately. Specifically, a variety of conventional diagnostic methods were utilized, including Blood cultures, sputum cultures, and smear preparations. Additionally, serological tests, including (1,3)-β-D-glucan detection (G-test) and *Mycoplasma pneumoniae* antibody detection, were also conducted. T-spot assay and GeneXpert should be used for the detection of *Mycobacterium tuberculosis*. Quantitative polymerase chain reaction (qPCR) technology was used to detect viral nucleic acids.

### Library preparation and sequencing

Pathogen DNA in patient’s samples was extracted using the TIANamp Magnetic DNA Kit (Tiangen) according to the manufacturer’s protocols. And DNA libraries were constructed by end repair, junction ligation and PCR amplification using the Hieff NGS C130P2 OnePot II DNA Library Prep Kit for MGI (Yeasen Biotechnology). Agilent 2100 Bioanalyzer and Qubit were used for quality control of the DNA libraries. Subsequently, qualified DNA libraries (50 bp, single-ended) were sequenced on the DIFSEQ-200 platform.

### Bioinformatics analysis

Following downstreaming, low-quality sequences, contamination from junctions, repeats, and shorter sequences (less than 36 bp in length) were eliminated from the raw data using Trimmomatic pre-processing, which produced high-quality sequencing data. Afterwards, Bowtie2 software was used to align and remove human host sequences from the human reference genome (hs37d5). The remaining non-human sequences were taxonomically classified using Kraken2 software against a custom microbial genome database (version 1.0.0). This comprehensive database incorporated the most recent bacterial, viral, fungal, and parasitic genomes sourced from GenBank (http://ftp.ncbi.nlm.nih.gov/genomes/genbank/).

### Interpretation of the mNGS report

The report reflects pathogenic and suspected background microorganisms based on species pathogenicity, and the causative organism is usually determined by a combined interpretation by 2 physicians based on the patient’s clinical signs (e.g., CT images, inflammatory markers).

### Statistical analysis

Based on the extracted data, 2 × 2 column tables were applied to determine the sensitivity and specificity of the mNGS, Fisher’s exact test was used to assess differences between qualitative variables, and the chi-square test was used to compare differences in positivity rates. Data were analysed and pictures drawn using SPSS 26.0 software and GraphPad Prism 8 software. All tests were two-tailed and *p*<0.05 indicated statistical significance.

## Results

### Baseline characteristics of patients

In this study, data on 205 patients were gathered, comprising 115 males (56.31%) and 90 females (43.69%). The ratio of male to female was about 1.3:1, and the age range was 14 to 92, with 60 being the median age. In patients with inflammatory markers, the mean value of WBC was 3.27*10^9^/L, which was lower than the normal range, while the mean value of CRP was 4.20mg/L, which was much higher than the normal range. Most of the patients had underlying diseases, hypertension accounted for the largest percentage (31.70%, 65/205), followed by diabetes (17.56%, 36/205) and pulmonary nodules (12.68%, 26/205), and 6 cases were in the postoperative state of the cancer (**Table 1**).

**Table 1:** Clinical information of the included population.

Characteristics	Patients with suspected LRTI (n =205)
Age, median [range], y	60 (14,92)
Female sex, n (%)	90 (43.90%)
Male sex, n (%)	115 (56.10%)
Laboratory results	
WBC count, median [range], 10^9^/L	9.115 [1.23-26.25]
PCT, median [range], ng/mL	3.0457 [0.027-54.56]
CRP, median [range], mg/L	79.11 [0.50-393.00]
hypersensitive CRP, median [range], mg/L	73.2828 [0.20-217.07]
Neutrophil %, media [range], %	77.0911 [40.0-96.9]
Underlying disease	
Hypertension, n (%)	65 (31.70%)
COPD, n (%)	25 (12.19%)
Cancer, n (%)	6 (2.93%)
Diabetes, n (%)	36 (17.56%)
Pulmonary nodules, n (%)	26 (12.68%)
bronchiectasis, n (%)	12 (5.85%)
Anemia, n (%)	16 (7.80%)

WBC, white blood cell; PCT, procalcitonin; CRP, C-reactive protein; COPD, Chronic Obstructive Pulmonary Disease.

### The overall detection of mNGS

**Figure 2:**
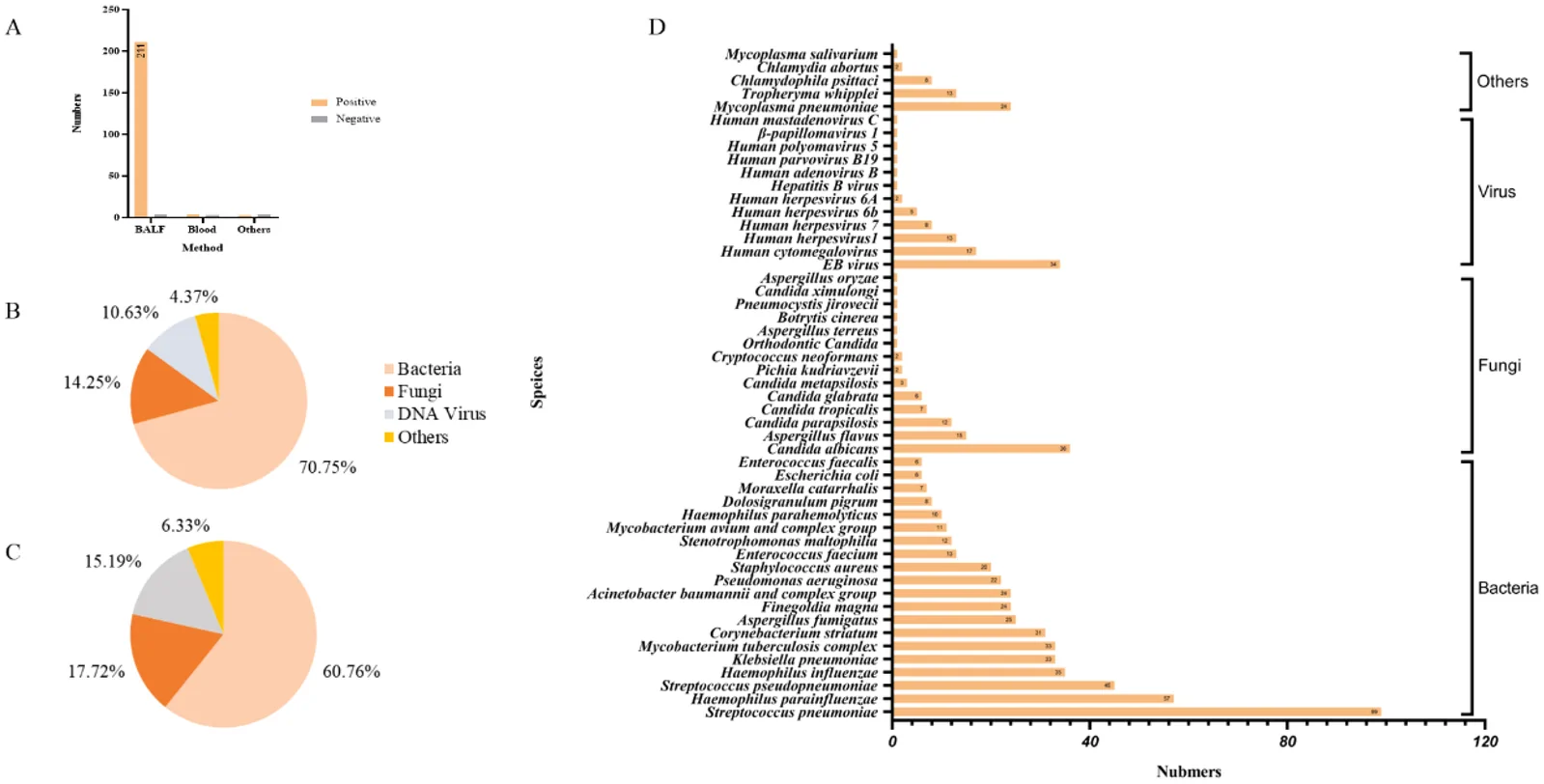
Pathogen detection based on mNGS. **(A)** The number of positive cases of different sample types; **(B)** The detection instances of different types of pathogens in mNGS; **(C)** The detection of different types of pathogens in mNGS species; **(D)** Pathogenic microorganisms reported by mNGS.

The overall assessment of mNGS was performed on 229 samples of 205 patients, including 215 cases of alveolar lavage fluid, 7 cases of blood and 7 cases of other samples (**Figure 2A**). A total of 79 different pathogens were found, and the overall positive rate was 95.20% (219/229). Bacteria were the most common pathogens, comprising 70.25% of the total detection instances and 60.76% of the total number of distinct species identified (**Figures 2B, C**). The five most prevalent bacteria were *Mycobacterium tuberculosis* (14.41%), *Klebsiella pneumoniae* (14.41%), *Haemophilus influenzae* (15.28%), *Streptococcus pneumoniae* (43.23%), and *Haemophilus parainfluenzae* (24.89%). Among the fungal pathogens, *Candida albicans* (15.72%), *Aspergillus flavus* (6.55%), *Candida parapsilosis* (5.24%), *Candida tropicalis* (3.06%) and *Candida glabrata* (2.63%) accounted for the highest proportion. Regarding viruses, except *EB virus* (14.85%), *Human cytomegalovirus* (7.42%), *Human herpesvirus 1* (5.68%), *Human herpesvirus 7* (3.49%), *Human herpesvirus 6B* (2.18%) and *Human herpesvirus 6A* (0.87%), other viruses were detected only once. Furthermore, *Mycoplasma pneumoniae* (10.48%) and *Tropheryma whipplei* (5.68%) also had the detection rates worthy of clinical attention (**Figure 2D**).

### Comparison of detection performance and consistency between mNGS and CMT results

**Figure 3:**
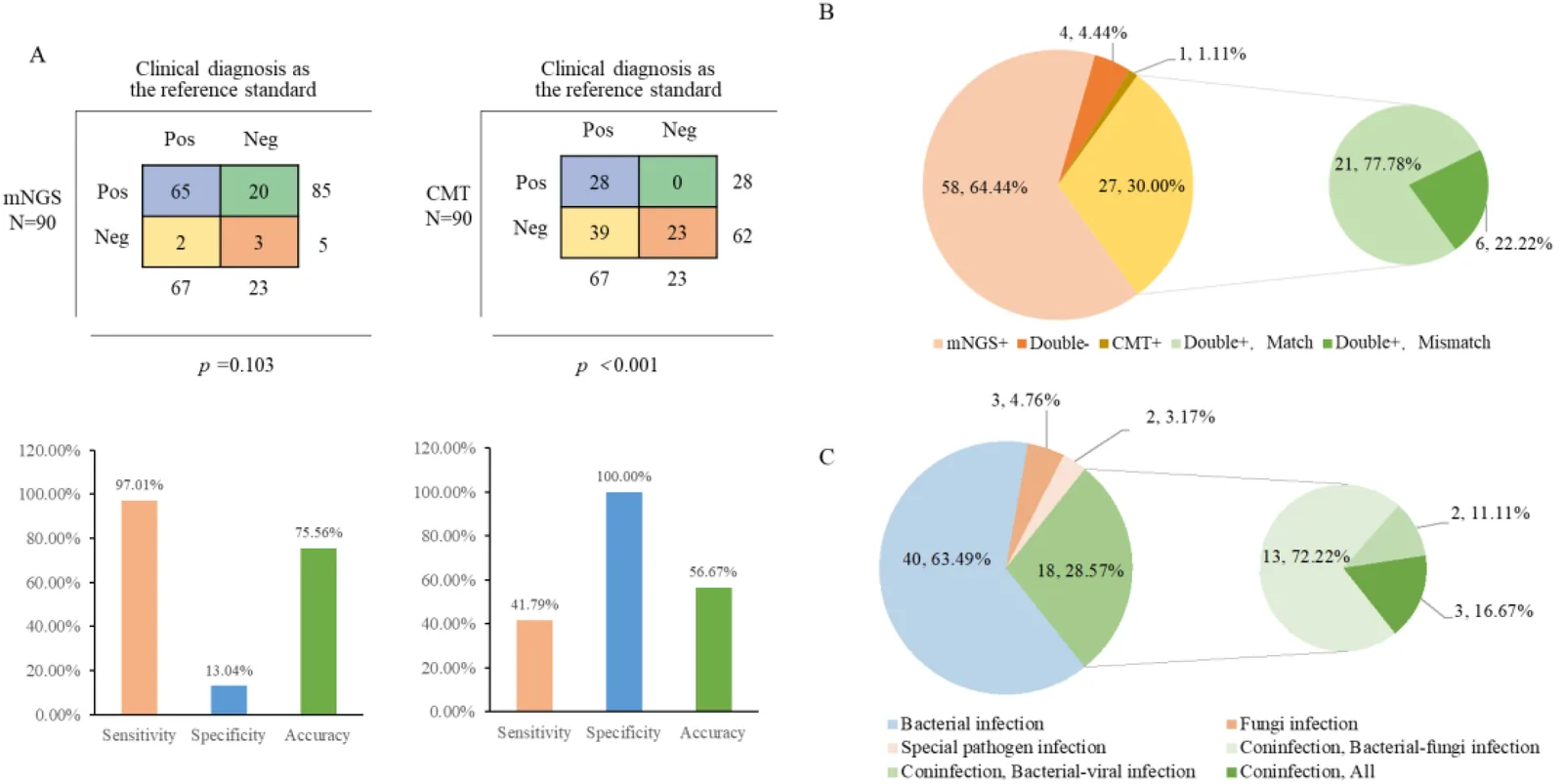
Comparison of results and consistency with cases of mNGS and CMT. **(A)** The 2×2 contingency tables comparing the performance of mNGS relative to CMT; **(B)** Comparison of Consistency between mNGS and CMT; **(C)** Diagnosis of infection types.

A total of 90 samples from 84 patients were subjected to traditional testing concurrently. Using the final “clinical diagnosis” as the gold standard, the sensitivity and accuracy of mNGS were found to be superior to those of traditional techniques, at 97.01% (65/67) and 75.56% (68/90), respectively. However, the specificity of mNGS was only 13.04% (3/23), while CMT exhibited a specificity of 100% (23/23) (**Figure 3A**). In terms of consistency, 27 samples (30%) were positive for both mNGS and CMT. In addition, 58 samples were detected solely by mNGS as potential pathogens, while one sample was positive only by CMT. The remaining four samples (4.4%) tested negative by both methods. Among the samples that were positive by both methods, 21 demonstrated a certain degree of consistency, while the remaining 6 cases could not be matched (**Figure 3B**). Most of the inconsistent samples revealed CMT fungal false positives, including one instance of *Klebsiella pneumoniae* and five cases of Candida (four instances of *Candida albicans* and one of *Candida tropicalis*). Additionally, mNGS identified two further cases of *Mycobacterium tuberculosis complex* infections. Out of the 84 patients, 63 were ultimately diagnosed with clear pathogenic information. The most prevalent type of infection was bacterial, accounting for approximately 63.49% (40/63) of cases, followed by mixed infections at 28.57% (18/63), with more than half of the mixed infections reflecting bacterial-fungal combinations (**Figure 3C**).

### The detection of mNGS pathogens and antibiotic resistance genes in COPD and Non-COPD group

**Figure 4:**
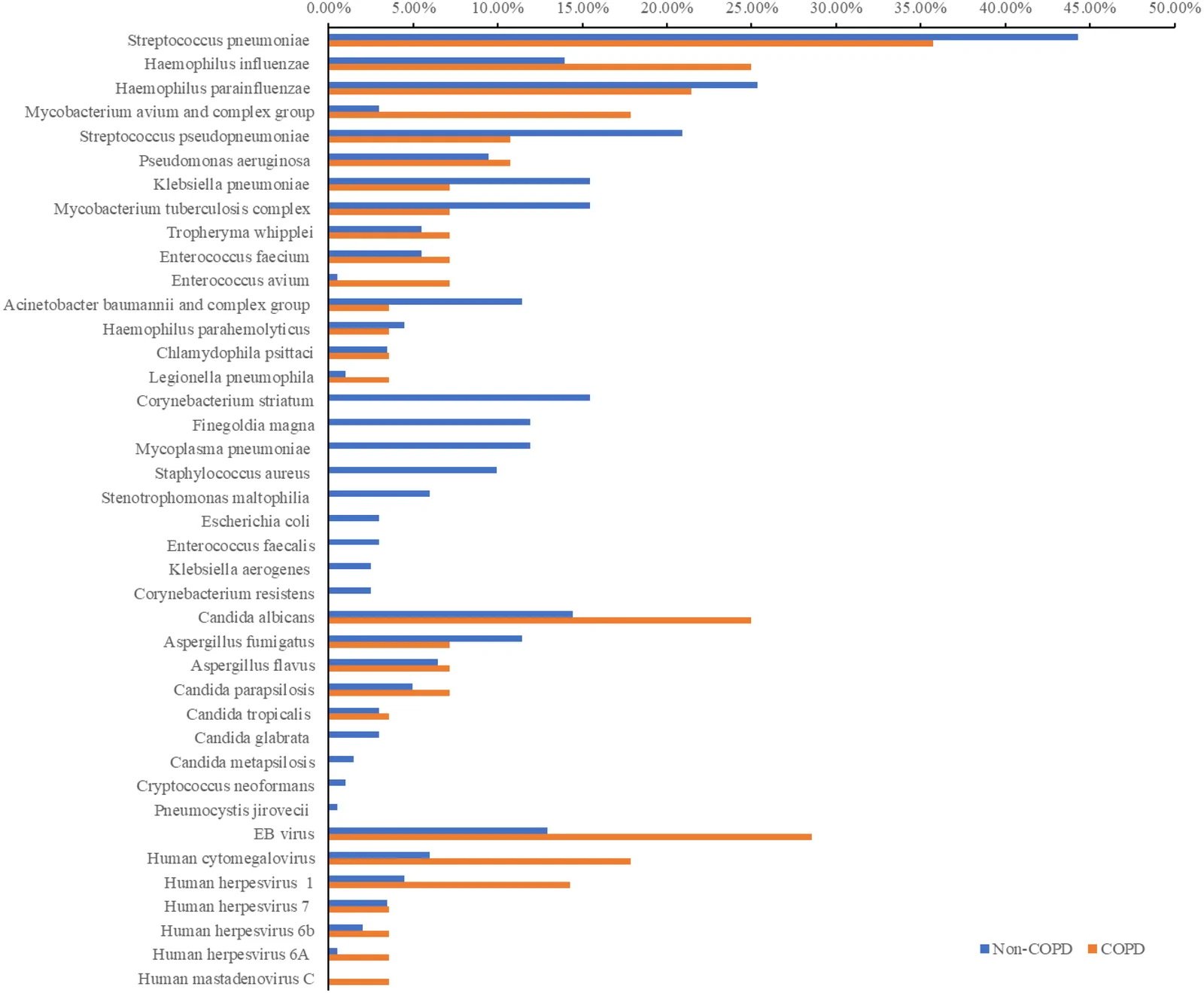
Pathogens of mNGS in COPD and Non-COPD group.

Patients were classified into two groups: those with COPD (28 cases) and those without COPD (201 cases), and the microbiological detection using mNGS was compared between both groups (**Figure 4**). *Streptococcus pneumoniae* (44.28% vs. 35.71%), *Candida albicans* (14.43% vs. 25.00%), and Epstein-Barr virus (12.94% vs. 28.57%) were the predominant bacteria, fungi, and viruses in both groups. Compared to the non-COPD group, the detection rates of *Haemophilus influenzae*, *Mycobacterium avium complex*, *Pseudomonas aeruginosa*, *Tropheryma whipplei*, and *Chlamydia psittaci* were higher in the COPD group. Conversely, *Corynebacterium striatum*, *Mycoplasma pneumoniae*, and *Staphylococcus aureus* were detected only in the non-COPD group. Notably, the detection rates of significant pathogens such as *Mycobacterium tuberculosis* (15.42% vs 7.14%) and *Aspergillus fumigatus* (11.44% vs 7.14%) were higher in the non-COPD group than in the COPD group. Furthermore, in terms of fungal and viral detection, the COPD group exhibited higher rates, particularly for *Candida albicans*, *Aspergillus flavus*, Epstein-Barr virus, Human cytomegalovirus, and Human herpesvirus.

Additionally, a thorough analysis of the detected antibioticresistance genes was performed, which originated from 6 COPD patients and 31 non-COPD patients (**Supplementary Table 1**). The most prevalent resistance genes across all patients included TetM (17, 8.29%), mel (6, 2.93%), and PC1 beta-lactamase (blaZ) (3, 1.46%). Notably, the COPD group uniquely exhibited the presence of TEM-183, PDC-5, and PDC-3.

### Analysis of related factors affecting the type of infection

The verified pathogen data separated it into two categories: single-species infections and multi-species infections, for which two individuals lacked immunity data, the total of 179 patients were eventually included in order to examine the potential effects of each variable on the type of infection. The multivariate binary logistic regression analysis model includes several significant univariate analysis indicators as independent variable X, such as gender, immunity, whether hypertension, whether COPD, and the dependent variable Y, which is the kind of infection. The kind of infection had no relationship with gender, hypertension, or COPD (*p*=0.679, *p*=0.229, and *p*=0.345), while the immune status (*p*=0.009) was statistically significant (**Table 2**).

**Table 2:** Summary of the multivariate binary logistic regression analysis for immunity, hypertension, and COPD predicting the kind of infection.

Variables	β	SE	Wald	P Value	Exp(B)	95%CI
Gender	0.134	0.323	0.172	0.679	1.143	0.607∼2.154
Immune status	-0.881	0.338	6.780	0.009	0.415	0.214∼0.804
Hypertension (or not)	-0.441	0.365	1.462	0.227	0.643	0.315∼1.315
COPD (or not)	-0.461	0.487	0.893	0.345	0.631	0.243∼1.640

## Discussion

In our study, bacterial infections were most prevalent, comprising 70.25% of identified species and detected in 60.76% of samples by mNGS. Viral detection was lower (10.63% of species, 15.19% of samples) due to the exclusive testing of DNA nucleic acids, which precluded identification of RNA viruses such as Influenza A, Influenza B, and Human orthopneumovirus. To enhance diagnostic comprehensiveness, clinicians should tailor metagenomic testing to patient-specific needs. Fungal species *Aspergillus flavus* and *Candida* were notable, reflecting increased hospital-acquired infection risks (Toth et al., 2017). Following the COVID-19 pandemic, opportunistic bacteria, fungi, and eukaryotic viruses have been shown to proliferate in the gut microbiota of infected individuals (Zuo et al., 2021). The host immunity is reduced, and these species overgrow, causing pulmonary fungal infections (Nobile and Johnson, 2015). Notably, mNGS also detected *Mycoplasma pneumoniae* (10.48%) and *Tropheryma whipplei* (5.68%). Clinically, IgM antibodies or PCR were used for early detection of *Mycoplasma pneumoniae*, and Immunohistochemistry and Periodic acid-Schiff (PAS) were used to routinely detect *Tropheryma whipplei* infection (Prudent et al., 2019), however both methods had limitations and produced false positives. mNGS can efficiently circumvent the laborious and time-consuming limitations associated with various targeted test procedures for all types of bacteria causing respiratory tract infections. It can also promptly give an etiological foundation, particularly for patients whose cultures are negative.

This study also evaluated the spectrum of pathogens and the identification of treatment resistance genes in populations with and without COPD. The detection frequencies of Epstein-Barr virus (EBV), human cytomegalovirus, *Candida albicans*, and *Haemophilus influenzae* were significantly higher in the COPD group. These findings are consistent with previous reports that EBV is the most frequently detected virus in stable COPD patients and *Candida albicans* is the most common fungal pathogen (Li et al., 2022). Additionally, the incidence of severe viral infection in acute exacerbations of COPD (AECOPD) was reported to be 41.2%, with Rhinovirus/Enterovirus being the most commonly detected virus (Jang et al., 2021). However, our study did not perform RNA extraction and detection, limiting our ability to verify these findings. EBV is known to persist in the body following pharyngeal infection, and most healthy individuals develop an adaptive immune response (Damania et al., 2022). High susceptibility to EBV has been implicated as a potential cause of frequent AECOPD (Geerdink et al., 2016). This susceptibility may be related to immune system changes, such as reduced frequencies of CD4+ central memory T cells and CD8+ effector memory T cells, which increase the risk of viral respiratory infections and subsequent exacerbations (Kim et al., 2016). When combined with bacterial infections, the clinical outcomes may be more severe. In this study, patients with a history of COPD were ultimately diagnosed with AECOPD.

For antibiotic resistance genes, the most common infections among all patients were tetM, mel, and blaZ. TetM is one of the most common resistance genes of extended-spectrum beta-lactamase. It has been found in *Enterococcus faecium*, *Listeria monocytogenes*, *Staphylococcus aureu*s, *Streptococcus pneumoniae* and other Gram-negative bacteria (Gay et al., 2000; Yıldız et al., 2014; Haubert et al., 2018; Dos Santos et al., 2021). The resistance genes of TEM-183, PDC-5 and PDC-3 were only detected in the COPD group, indicating potential resistance of *Haemophilus influenzae* and *Pseudomonas aeruginosa* to antibiotics, including monobactams, carbapenems, and cephalosporins. These medications are frequently used as broad-spectrum therapeutic antibiotics. As detection technology continues to advance, the use of mNGS in drug resistance gene identification can expand clinical practice’s diagnostic foundation. If the corresponding antibiotic resistance genes are detected, their pathogenicity should be considered and supplemented by other means, so that mNGS can play more roles in drug resistance gene detection and treatment drug selection.

We observed that traditional methods have limited ability and lower sensitivity in detecting a wide range of pathogenic microorganisms in the study. In contrast, mNGS offers the advantage of identifying a broad spectrum of microbes in LRTIs patients, including previously recognized pathogens and atypical microorganisms of uncertain significance. However, the specificity of mNGS was relatively low, though consistent with some previous studies (Diao et al., 2023). This may be attributed to the inherent detection principles of mNGS as a non-targeted, high-throughput sequencing technology, which detect all microbial nucleic acids present in a sample, including non-pathogenic microorganisms, colonizers, commensal, and potential contaminants (Miao et al., 2022; Lv et al., 2024). Consequently, while mNGS can identify true pathogens, it may also misidentify non-pathogenic microorganisms as sources of infection, thereby generating false-positive results. Therefore, it is crucial to combine mNGS results with the epidemiological and clinical characteristics of patient to accurately identify pathogens and guide effective treatment.

There are limitations to this study. Firstly, the sample size used to evaluate detection performance and consistency may limit the reliability of our findings, and the restricted variety of specific sample types could compromise the robustness of our statistical analyses. Additionally, during the extraction of pathogenic nucleic acids from the sample, human nucleic acids are also present, which may interfere with the detection of certain species. Furthermore, the analysis of drug resistance genes in COPD and non-COPD patients lacked a prospectively matched cohort of COPD patients. However, it is encouraging that mNGS still shows positive diagnostic value, and further large-scale prospective studies are warranted to explore the value of mNGS in rapid and accurate diagnosis, as well as for assessing drug resistance and virulence information.

## Conclusion

This study demonstrates the great potential of mNGS in the diagnosis of pathogens in patients with clinically suspected lower respiratory tract infections. When compared to conventional techniques, mNGS exhibits superior timeliness, accuracy, and sensitivity. Multiple microorganisms could be identified simultaneously for unusual or hard-to-identify pathogens. mNGS helps identify mixed infections and stops the development of bacterial resistance, provided a substantial and vast knowledge base for guiding clinical focused therapy. Consequently, mNGS is a promising quick molecular diagnostic method that should not be disregarded.

## Data Availability

All sequence reads were deposited into the Genome Warehouse in the National Genomics Data Center under the accession number PRJCA035097.
