# Correction: The application prospect of metagenomic next-generation sequencing technology in diagnosing suspected lower respiratory tract infections

**DOI:** 10.3389/fcimb.2026.1949477

**Published:** 2026-08-10

**Authors:** Wei Li, Mingming Zhao, Weiwei Wu, Gang Chen, Yanping Hang, Haixia Zheng, Zhenyun Gao, Jia Liu, Yuguo Zhao

**Affiliations:** 1 Department of Respiratory Medicine, Nanjing Lishui People’s Hospital, Zhongda Hospital Lishui Branch, Southeast University, Nanjing, Jiangsu, China; 2 Department of Pulmonary and Critical Care Medicine, Nanjing Gaochun People’s Hospital, Nanjing, Jiangsu, China; 3 Dinfectome Inc., Nanjing, Jiangsu, China

**Keywords:** suspected lower respiratory tract infection, metagenomic next-generation sequencing, COPD, pathogen spectrum, resistance genes

Authors “Yuguo Zhao” and “Jia Liu” were erroneously assigned as equal contributing author.

The original version of this article has been updated.

