# Supplementary material for: The application prospect of metagenomic next-generation sequencing technology in diagnosing suspected lower respiratory tract infections
Source: Front Cell Infect Microbiol. 2025 Apr 16;15:1494638. doi: 10.3389/fcimb.2025.1494638 (PMC12041802; doi:10.3389/fcimb.2025.1494638)
Supplement: Supplementary file 1 [file Table1.docx]

Supplementary Table 1 Detection of drug resistance genes in COPD and non-COPD patients

| Group | Sample | Drug resistance genes |
| --- | --- | --- |
| COPD | Patient 1 | mel\|tetM |
|  | Patient 2 | TEM-183 |
|  | Patient 3 | PDC-5 |
|  | Patient 4 | PDC-3 |
|  | Patient 5 | tetM |
|  | Patient 6 | tetM |
| Non-COPD | Patient 1 | sul1 |
|  | Patient 2 | ANT(9)-Ia\|mecA\|PC1 beta-lactamase (blaZ) |
|  | Patient 3 | CrpP |
|  | Patient 4 | mel\|SHV-24 |
|  | Patient 5 | APH(3')-IIIa |
|  | Patient 6 | SHV-12 |
|  | Patient 7 | tetM |
|  | Patient 8 | APH(6)-Id |
|  | Patient 9 | tetM |
|  | Patient 10 | PC1 beta-lactamase (blaZ) |
|  | Patient 11 | tetM |
|  | Patient 12 | tetM |
|  | Patient 13 | tetM |
|  | Patient 14 | LEN-8\|mel |
|  | Patient 15 | LEN-23 |
|  | Patient 16 | Mycobacterium tuberculosis rpsL mutations conferring resistance to Streptomycin |
|  | Patient 17 | tetM |
|  | Patient 18 | SHV-21\|SHV-41 |
|  | Patient 19 | CTX-M-50 |
|  | Patient 20 | tetM |
|  | Patient 21 | mel\|tetM |
|  | Patient 22 | tetM |
|  | Patient 23 | tetM |
|  | Patient 24 | OXA-103 |
|  | Patient 25 | tetM |
|  | Patient 26 | mel\|Mycoplasma pneumoniae 23S rRNA mutation conferring resistance to erythromycin |
|  | Patient 27 | mel |
|  | Patient 27 | PC1 beta-lactamase (blaZ) |
|  | Patient 28 | tetM |
|  | Patient 29 | tet(K) |
|  | Patient 30 | tetM |
|  | Patient 31 | aad(6)\|catB3\|tetM |
